# Flexural Creep Behaviour of Pultruded GFRP Composites Cross-Arm: A Comparative Study on the Effects of Stacking Sequence

**DOI:** 10.3390/polym14071330

**Published:** 2022-03-25

**Authors:** Abdulrahman Alhayek, Agusril Syamsir, Abu Bakar Mohd Supian, Fathoni Usman, Muhammad Rizal Muhammad Asyraf, Mohd Afdzaluddin Atiqah

**Affiliations:** 1 Civil Engineering Department, College of Engineering, Universiti Tenaga Nasional, Kajang 43000, Malaysia; rahman.hayek@gmail.com (A.A.); fathoni@uniten.edu.my (F.U.); 2 Institute of Energy Infrastructure (IEI), College of Engineering, Universiti Tenaga Nasional, Kajang 43000, Malaysia; asyrafriz96@gmail.com; 3 Institute of Microengineering and Nanoelectronics, Universiti Kebangsaan Malaysia, Bangi 43600, Malaysia; a.atiqah@ukm.edu.my

**Keywords:** stacking sequence, pultrusion, creep, glass fibres-reinforced polymer, flexural, cross arm

## Abstract

Pultruded glass fibre reinforced polymer (pGFRP) composites provide outstanding properties for composite polymer cross arms in power transmission line applications. This study has investigated the effects of various stacking sequences of fibres directions of pGFRP on flexural strength and creep behaviour. The use of static four-point bending flexural tests revealed that Stacking Sequence 2 (±45/0/90/0/90/0) had a significant flexural strength of 399.9 MPa while Stacking Sequence 1 (±45/90/0/±45) had a flexural strength of 242.5 MPa. Furthermore, the four-point bending creep experiments were performed at three distinct stress levels, notably 12%, 24%, and 37% of the ultimate flexural strength, to characterise the creep behaviour of distinct stacking sequences. Moreover, Findley’s power law equation for bending creep behaviour has revealed that the time-dependent reduction factor of Stacking Sequence 1 and Stacking Sequence 2 estimates a drop in flexural modulus of 23% and 10% respectively.

## 1. Introduction

An overhead power line transmission tower, also known as a power tower or an electricity pylon, is generally a large structure made of steel lattice that supports the line. Generally, 132 kV, 275 kV, and 500 kV are the main electrical cable types used in power line transmission tower systems [1,2]. Over time, polymeric materials have replaced wooden cross arms in transmission towers due to several factors, such as the drastic decline of timber resources and cost-effectiveness [3,4,5]. Due to its non-conductivity and high dielectric strength, the pultruded glass fibre reinforced polymer (pGFRP) cross arm serves as a surplus component for an insulator, where the combined insulation improves the lightning impulse voltage performance of an electrical power line’s distribution and transmission line construction. Simultaneously, the pGFRP cross arm has evolved to withstand transmission line assemblies, however, one of the difficulties linked with these materials’ behaviour in fabrication is its high tendency towards creep effects.

Creep is defined as a time-dependent plastic deformation that occurs at elevated temperatures and with constant stress lower than the yield stress. However, while polymers exhibit a similar time-dependent deformation that is viscoelastic in nature, their behaviour presents the characteristics of elastic solids [6,7,8,9,10]. Meanwhile, the creep phenomenon of FRP is significant because it can endanger the dependability and durability of structural components and have a tendency to buckle permanently as a result of mechanical pressures caused by long-term interaction with high levels of stress [10,11,12,13]. Although there are various fabrication processes for producing GFRP structures, the pultrusion method was extensively used to produce hollow tubes, such as the pGFRP cross arm. This is because using the pultruded composite method to create pGFRP cross arm structures has many advantages, such as strength, lightness, stiffness, easy and rapid installation, anti-corrosion, and acoustic insulation [14,15,16]. The pultrusion method enables the fabrication of prismatic profiles in which fibre orientation is optimised and orientated longitudinally [17,18]. Furthermore, the mechanical characteristics of pGFRP composites in the pultrusion technique are influenced by various factors, such as fibre volume, interphase matrix, fibre orientation, stacking sequence, and others [19,20].

**Figure 1:**
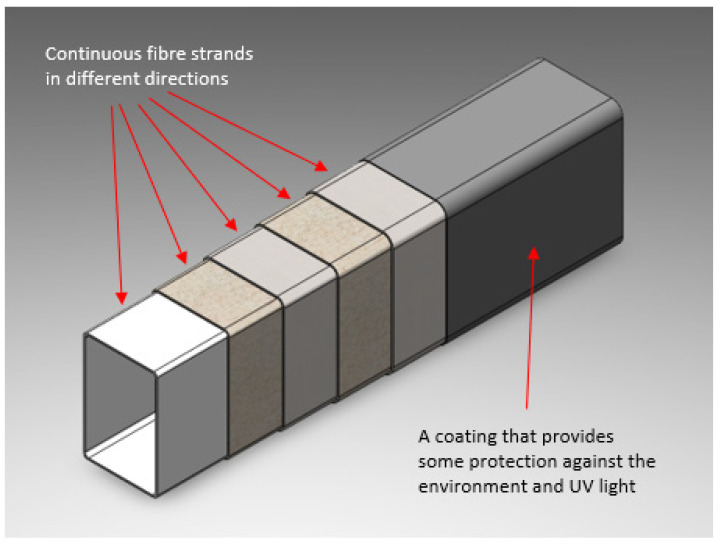
Cutaway illustration of pGFRP cross arm.

The advantages of composite cross arms are that they provide robust performance. Because of this, they are used as anchor points, designed to replace wooden structures at the dead-end assemblies within transmission and distribution systems. Likewise, pGFRP cross-arms have been employed as cantilever beams for street light support structures as they are a good choice when supplying arm supports for attaching outdoor components to utility poles [2,21]. However, pGFRPs are often subject to extreme outdoor exposure and constant loads for extended periods of time, resulting in a behaviour known as structural collapse, which occurs due to creep deformation. Therefore, creep behaviour is a critical issue for materials that have endured loads for extended periods of time [2,22,23,24]. The study by Beddu et al. [25] of the creep behaviour in GFRP cross arms indicates that the initial creep response causes the cross arm to change size and shape when subjected to long-term loading. An illustration of a cutaway cross arm is shown in Figure 1.

The biaxial fibre stacking sequence pultrusion technique is used in pGFRP cross arms because it integrates different forms of oriented fibres; biaxially and oriented polymeric fibres/fillers inside the matrix interact with each other as load-bearing components, an effect comparable to incorporating several materials in of woven textiles [20,26]. The fibre stacking sequence plays a significant part in the enhancement of mechanical properties. As such, woven textiles are one of the most effective reinforcing structures for facilitating physical interferences within reinforced fibre and matrix systems. Furthermore, the stacking sequence contributes significantly to a change in the mechanical characteristics and performance of pGFRP cross arms in transmission tower construction [19,27]. While investigating the effects of stacking sequences upon various mechanical properties and the failure mode responses of quasi-static compression loads [27,28,29,30], numerous computational models were considered. However, the four-element burgers (FEB) model and the Findley power law (FPL) model are among the most prevalent [1,8,31,32,33]. 

While there are other models to describe the behaviour of GFRP when subjected to creep, Findley’s power law model has proven to be reliable and accurate, in addition to being easy and simple to use [8,31]. Hence, this paper will use Findley’s power law to model the behaviour of pGFRP and predict losses in stiffness due to creep. The aim of this study is first to determine the ultimate flexural strength of each sequence by using four-point bending tests to calculate the respective creep load levels, then perform the creep tests. We studied two pGFRP cross arm sequence layouts, Stacking Sequence 1 (±45/90/0/±45) and Stacking Sequence 2 (±45/0/90/0/90/0), with a total thickness of 6 mm and 7 mm, respectively. The coupons produced via the pGFRP cross arms have been subjected to four-point bending creep tests for a period of 720 h at three different load levels for each respective sequence. Finally, Findley power law parameters were obtained to highlight the difference in creep performance between the sequences and calculate the reduction factor. 

## 2. Material and Methodology

### 2.1. Cross Arm Fabrication

**Figure 2:**
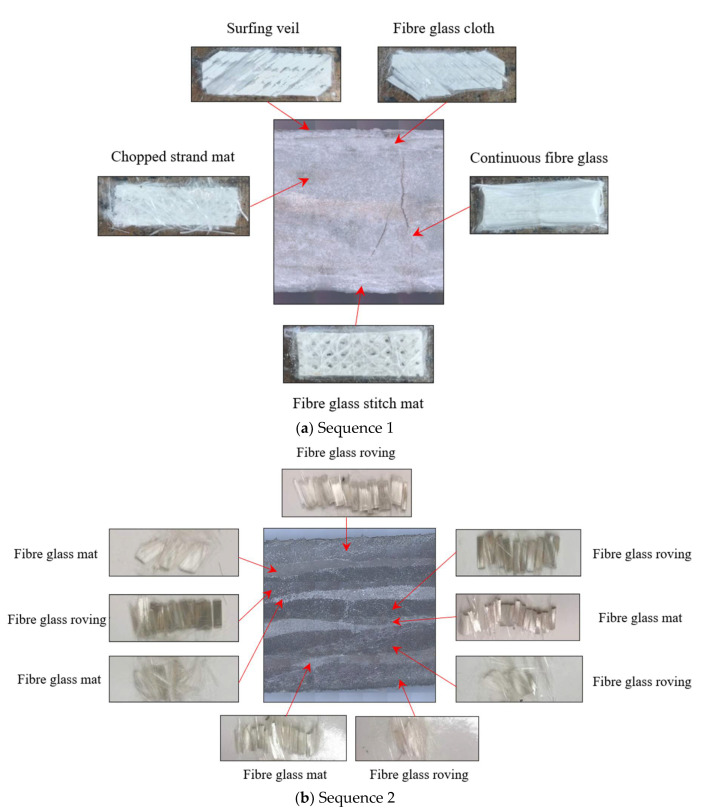
Breakdown of fibres plies in pGFRP cross-section: (**a**) Sequence 1; (**b**) Sequence 2.

Pultrusion is a continuous manufacturing process for stable cross-section composite materials. The pGFRP cross arm has been integrated with a high-strength fibreglass mat, which, when merged with a reinforced fibreglass strand, provides multi-directional reinforcement and resistance. In other words, unidirectional fibres are arranged in layers separated by continuous strand-mats, as illustrated in Figure 2. These components are integrated and immersed in a liquid resin mixture before being pulled through a hot steel-forming die. As a result, a robust, fibreglass-reinforced composite structure is created that will never deteriorate, unlike wood. The coupon samples used for the testing were taken from the wall segments of hollow tubes of the pGFRP cross arm, which were manufactured via the pultrusion technique by various vendors. The pGFRP hollow tubes were acquired from two different sequences of fibre arrangements and layered with distinct stacking sequences. Both sequences use polyester resin and glass fibres with a fibre volume fraction, $V_{f}$, of 62% and 70% for Sequence 1 and Sequence 2, respectively, while the remaining volume fraction, 100%, is the used resin. Table 1 presents the material properties in each sequence. The fibre direction plies/stacking sequence of the samples is described as the following sequence:

Sequence 1: (±45/90/0/±45) with a thickness of 6 mm.

Sequence 2: (±45/0/90/0/90/0) with a thickness of 7 mm. 

A total of six replicate coupon samples for each stacking sequence were cut from pGFRP hollow tubes with the same dimensions of 38 mm × 380 mm. Three samples were tested in static failure four-point bending tests, and the remaining three were used in flexural creep tests at three different load levels, with one specimen at each load level. 

### 2.2. Fibre Volume Fraction

The stacking sequence parameter becomes one that contributes to the quantity of resin to fibre ratio while producing the pGFRP composite cross arm. As a result, the ASTM D2584 [34] was utilised as a guideline to analyse the variations in fibre volume fraction, which impacts the amount of resin that may be absorbed by fibres. One of the elements influencing the general mechanical characteristics of composite structures is the fibre percentage of fibre reinforcement in stacking sequence composite structures [27,35]. The pultrusion technique’s customised design of the stacking sequence at various interlaminar of glass fibre incorporation has impacted diversity, strength, and behaviour.

**Figure 3:**
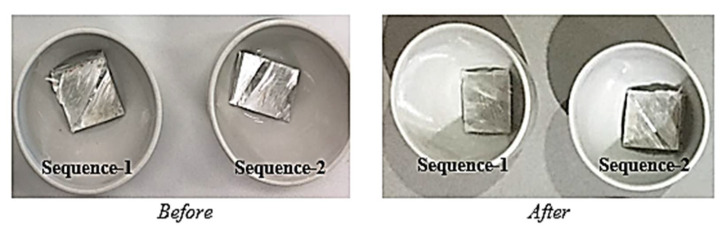
Burn-off test of pGFRP specimen.

The pGFRP tubes were cut into 20 mm × 20 mm pieces and subjected to burn-off tests in accordance with the ASTM D2584 standard to measure the fibre volume fraction. Using an electric oven (furnace), the specimens were heated to 600 °C for 1 h for total burning of the resin or other substances. Then, the residual glass fibres were sorted and precisely weighed, resulting in the left-over of fibre fractions, which are shown in Table 1, while the burn-off test specimens are shown in Figure 3.

### 2.3. Static 4-Point Bending Tests

**Figure 4:**
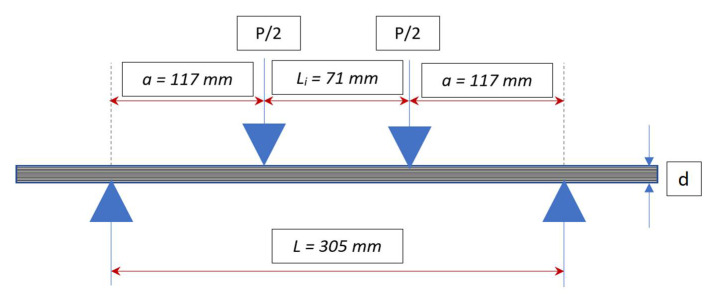
Schematic diagram of the four-point bending experimental setup.

**Figure 5:**
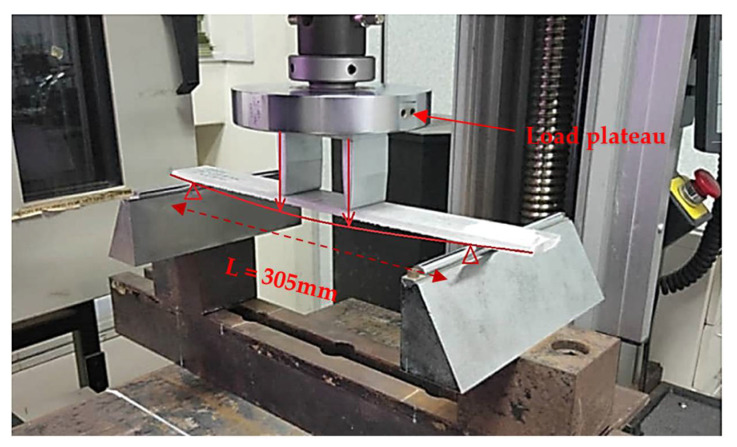
The static four-point bending test.

Quasi-static four-point flexural failure tests were performed following the guidelines of the ASTM D6272 standard to evaluate the flexural characteristics of the pGFRP laminates [36]. The test configuration, as shown in Figure 4, had a support span of 305 mm to ensure that failures only occurred in the outer fibres of the specimens, while the load span was 71 mm. The quasi-static load speed was set at 3.5 mm/min to ensure that the specimen would fail without exhibiting catastrophic behaviour (Figure 5). Then, using elastic beam theory [37], stresses and strains were estimated, as illustrated in Equations (1) and (2).

(1) $$\sigma =\frac{3P\left(L-L_{i}\right)}{2bd^{2}}$$

(2) $$\varepsilon =\frac{6\left(L-L_{i}\right)d\Delta }{4a^{3}-3aL^{2}}$$

where σ is stress in the outer fibre in (Mpa), *P* is the load in (N), *L* is the support span in (mm), $L_{i}$ is the loading span in (mm), *b* is the specimen width in (mm), *d* is the specimen thickness in (mm), Δ is the midspan deflection in (mm), and *a* is the distance from the support to the nearest loading point in (mm).

### 2.4. Flexural Creep Test

**Figure 6:**
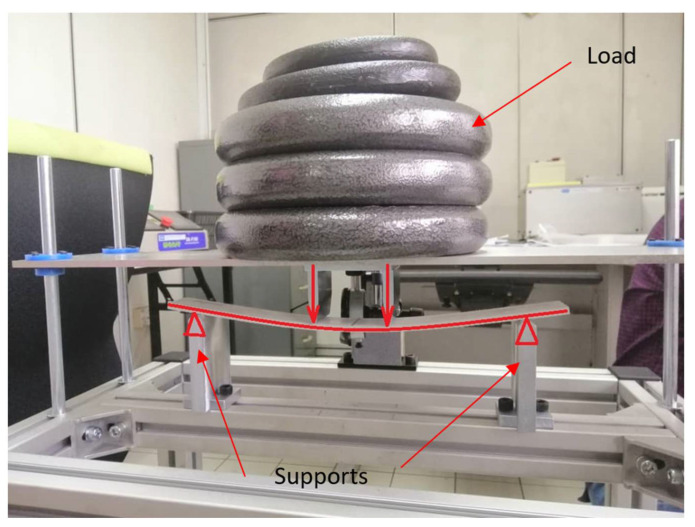
A coupon sample under four-point bending creep test.

The creep test arrangement was comparable to the ultimate flexural test system shown in Figure 4, with equivalent dimensions for loading and support spans. These tests followed the guidelines set by the ASTM D2990-17 standard [38]. Three load levels were set, namely 12%, 24%, and 37% of the ultimate flexural load. The experiments were conducted at room temperature for 720 h to allow creep to progress to the second stage. During the test, mid-span deflection was recorded immediately after loading, i.e., at time 0, and then every 15 min for the first 6 h. It was then taken every 24 h until the test was completed. The strain was determined using elastic beam theory after measuring the mid-span deflection with a dial gauge [37]. Figure 6 depicts a coupon specimen undergoing a creep test. 

Later, Findley’s method was used to determine the time-dependent reduction factor, which in turn was used to estimate the drop in modulus of elasticity using the parameters *n* and *m* for each sequence. According to the original form of Findley’s power law, total strain consists of an elastic time-independent component and a viscous time-dependent component, as indicated in Equation (3).

(3) $$\varepsilon \left(t\right)=\varepsilon_{o}+m\times t^{n}$$

where $\varepsilon_{0}$ is the instantaneous elastic strain, *t* is the time under sustained load (hours), *n* and *m* are material-specific parameters. 

## 3. Results and Discussions

### 3.1. Static Four-Point Bending Test Results

**Figure 7:**
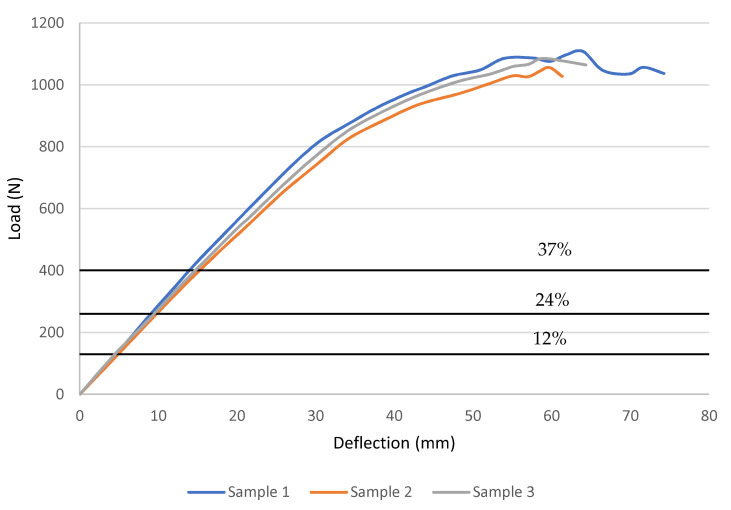
Flexural load vs. deflection curves from the failure tests for Sequence 1.

**Figure 8:**
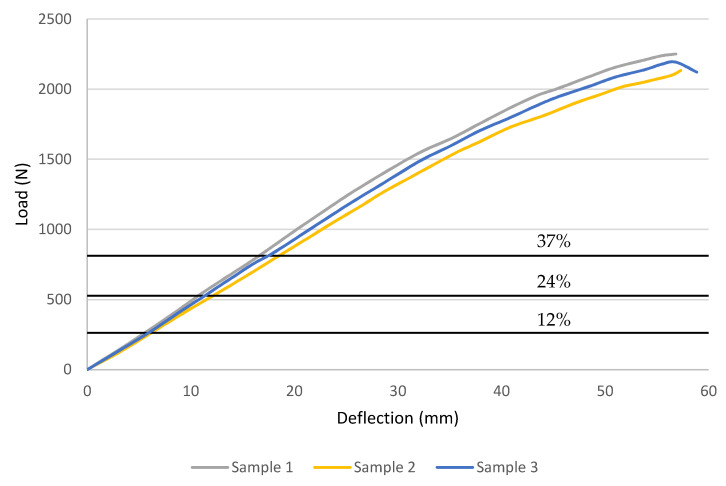
Flexural load vs. deflection curves from the failure tests for Sequence 2.

As previously discussed, short-term properties are important to determine the loads for the creep tests as they are taken as a percentage of the ultimate flexural strength. Three samples of each sequence were evaluated, with load versus deflection data displayed in Figure 7 and Figure 8, with horizontal lines denoting creep load levels. Meanwhile, Figure 7 and Figure 8 reveal that both stacking sequence specimens had a similar reaction, which, from the graph, exhibited linear elastic behaviour before failing due to brittleness. Table 2 summarises the stress and strain calculations. This behaviour is expected as GFRP laminates are not considered ductile materials, which is mainly attributed to the glass fibres in composites. For Sequence 1, the specimens had an average ultimate load of 1082 N with an average deflection at rupture of 66.6 mm, corresponding to an ultimate stress of 242.6 MPa and an ultimate strain of 0.02289 mm/mm. On the other hand, Sequence 2 exhibited a significantly higher strength where it recorded an average ultimate load of 2192 N with an average deflection at rupture of 57.7 mm, corresponding to an ultimate stress of 399.05 MPa and a strain at rupture of 0.02197 mm/mm. 

GFRP laminates show a clear dependency on fibre orientation and stacking sequences in their mechanical properties. In addition, damage and failure modes are influenced by the stacking sequence. However, symmetric and asymmetric stacking sequences displayed negligible differences [39,40,41].

### 3.2. Flexural Creep Results

**Figure 9:**
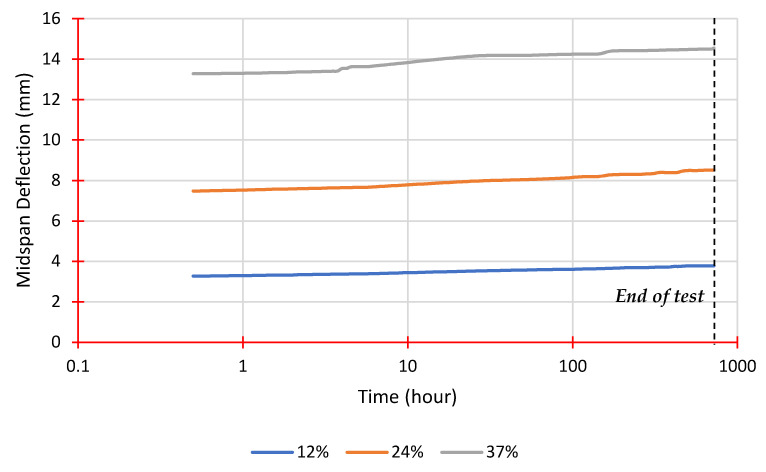
Midspan deflection vs. time from creep tests for Sequence 1.

**Figure 10:**
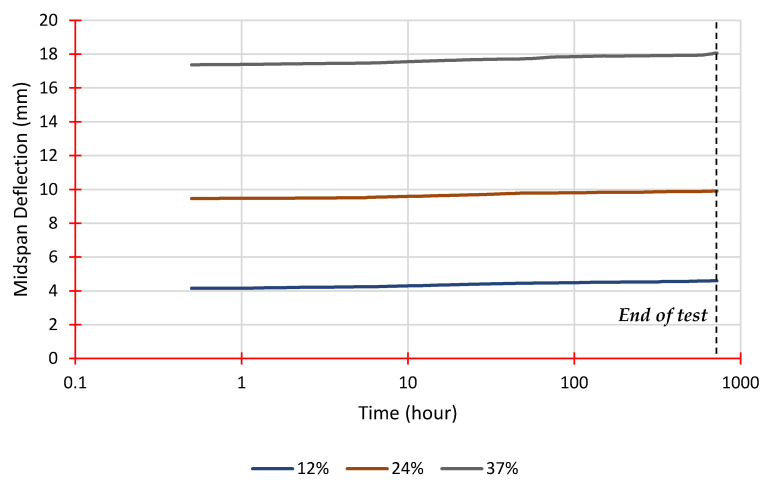
Midspan deflection vs. time from creep tests for Sequence 2.

The findings of the four-point flexural creep testing will be presented and discussed in this section. Table 3 shows the load levels and associated applied loads for each stacking sequence, which were calculated using the average ultimate flexural load obtained from static failure tests. Figure 9 and Figure 10 illustrate the midspan deflection as it rises over time for each load level for both stacking sequences. According to Figure 9, the primary stage of creep lasts longer with increasing load values, as described by Harries et al. [42]. Sequence 2, on the other hand, has higher flexural strength and flexural modulus than Sequence 1, which has a less pronounced trend, as seen in Figure 10.

As previously discussed, other researchers have already shown the effects stacking sequences can have upon the mechanical properties of GFRP laminates [43,44]. As such, we expected creep performance to be affected. What these results illustrate are the significant differences between the two stacking sequences.

The difference in the primary stage of creep between both stacking sequences suggests that the higher fibre volume fraction of 69.04% in Sequence 2 may have contributed towards lowering its duration since it has less resin, which is considered the main contributor for creep in GFRP, especially in the primary stage [43]. This emphasises the importance of a greater fibre volume percentage in increasing the member’s strength, stiffness, and resistance to creep.

**Figure 11:**
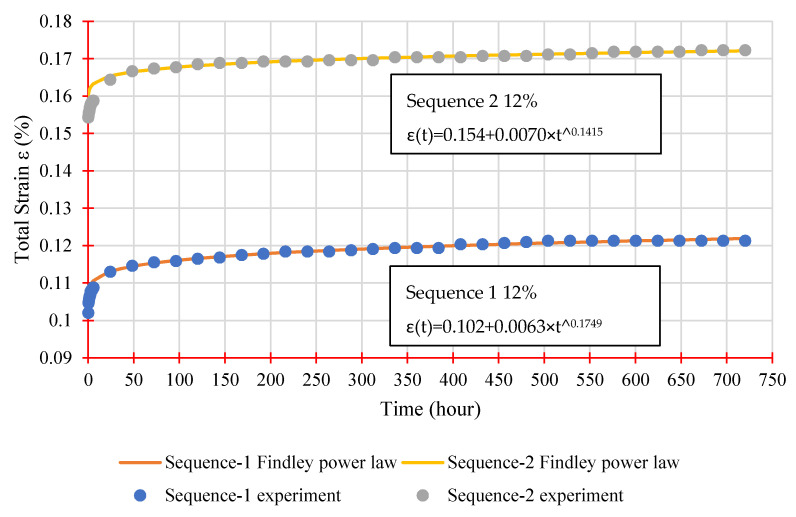
Findley’s power law parameters evaluation.

Findley’s power law, as illustrated in Equation (3), is used to examine creep behaviour in the primary and secondary stages. Findley’s model correctly describes the creep behaviour of FRP materials subjected to steady stress over an extended time range, while also being simple and straightforward to execute. The curve fits for both sequences at a 12% load level are shown in Figure 11, while Table 4 summarises all parameters derived from experimental data fittings.

As shown in Table 4, parameter *n* remains nearly constant for each sequence regardless of stress level, averaging 0.1724 and 0.1353 for Sequence 1 and Sequence 2, respectively. This is to be expected, given that previous studies have demonstrated that *n* is stress independent. The *m* parameter, on the other hand, is stress-dependent and rises with increasing stress levels, with an average of 0.0114% for Sequence 1 and 0.0084% for Sequence 2. Furthermore, both metrics, 0.12–0.35 for *n* and 0.0061–0.0184% for *m*, are within the ranges reported by other studies using similar stress levels. A generic Findley’s equation may be expressed below by taking the average value of *n* and *m* for each series.

(4) $$\mathrm{Sequence}\;1:\;\varepsilon \left(t\right)=\varepsilon_{o}+0.0114\times t^{0.1724}$$

(5) $$\mathrm{Sequence}\;2:\;\varepsilon \left(t\right)=\varepsilon_{o}+0.0084\times t^{0.1353}$$

**Figure 12:**
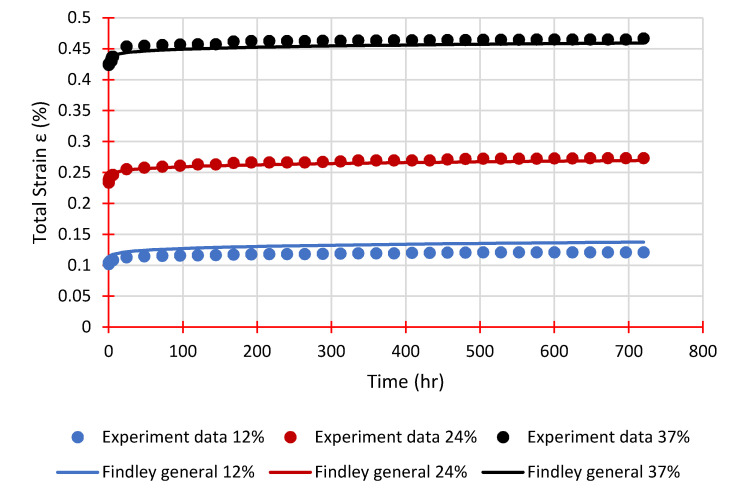
Experimental strains and Findley’s general equation for Sequence 1.

**Figure 13:**
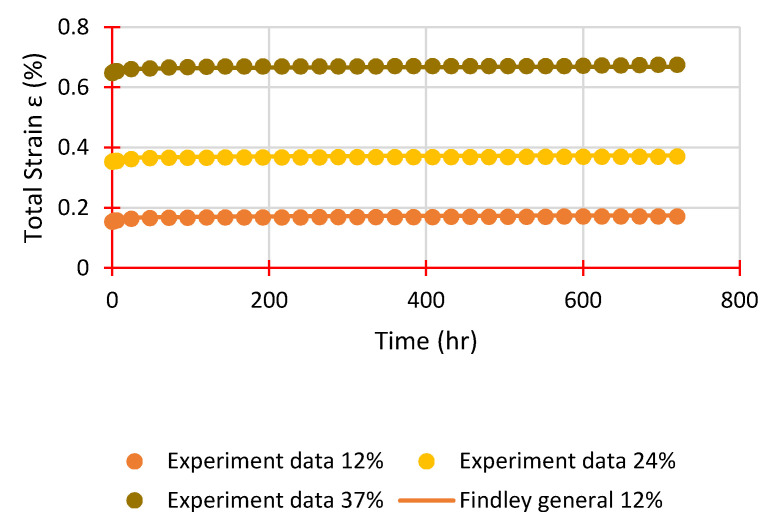
Experimental strains and Findley’s general equation for Sequence 2.

Equations (4) and (5) are plotted in Figure 12 and Figure 13 with the instantaneous elastic strain taken from the experimental tests. These equations are very closely matched with the measured data confirming the efficaciousness of Findley’s power law to simulate the creep behaviour of GFRP laminates. 

For the verification of the serviceability limit state, specifically long-term deformation, it is useful to obtain a reduced modulus of elasticity to be used in manual calculations or in an analysis program. Noting that $\varepsilon_{0}=\sigma /E_{0}$ and $m=\sigma /E_{t}$, Equation (3) can be rewritten in the following form:

(6) $$\varepsilon \left(t\right)=\frac{\sigma }{E_{o}}+\frac{\sigma }{E_{t}}\times t^{n},\;\varepsilon \left(t\right)=\frac{\sigma }{E\left(t\right)}$$

From Equation (6), the time-dependent flexural modulus can be obtained as follows:

(7) $$E\left(t\right)=\frac{E_{o}\times E_{t}}{E_{t}+E_{o}\times t^{n}}$$

Following a similar approach to Scott and Zureick [44], Equation (7) can be rearranged to have a time-dependent reduction factor χ(t).

(8) $$E\left(t\right)=E_{o}\times \chi \left(t\right)$$

(9) $$\chi \left(t\right)={\left(1+\frac{E_{o}}{E_{t}}\times t^{n}\right)}^{-1}$$

**Figure 14:**
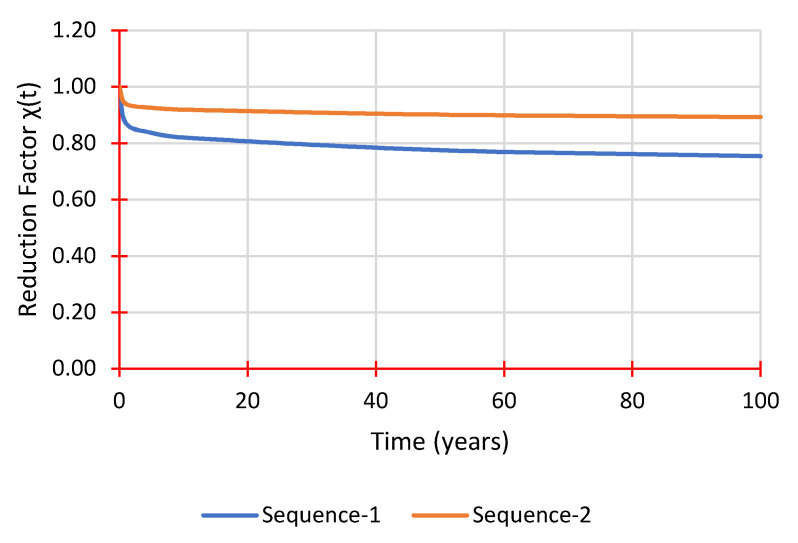
Reduction factor χ(t) prediction over time.

Equation (7) calculates the projected reduction in flexural modulus. Table 5 shows the flexural modulus for each sequence, determined using Equations (8) and (9) to calculate the expected reduction factors over numerous years. A significant variation between the two sequences is apparent, with Sequence 1 losing 23% of its rigidity and Sequence 2 losing only 10%. This highlights the importance of testing samples from a specific manufacturer to get tangible results, enabling more accurate modelling for GFRP laminates in the analysis and design stages. Moreover, one month of creep testing seems to be sufficient in providing creep material parameters via Findley’s power law as long as the stress level is not too high, to stay within the secondary stage of creep. Figure 14 shows the reduction factor over time in a graphical plot.

The results in Table 5 and Figure 14 show large discrepancies between different fibre stacking sequences that can be found in pGFRP cross arms and how sensitive the material characteristics and parameters are. On the other hand, it is clear that 90% of the initial flexural stiffness can be maintained over 50 years which is evidently influenced by the stacking sequence of pGFRP laminate.

## 4. Conclusions

An experimental investigation of the flexural creep behaviour of glass fibre reinforced polymer laminates made by pultrusion is discussed in this study. Two different sequences were used to highlight the differences in the material properties’ creep behaviour. The short-term and long-term tests had the same four-point bending configuration and dimensions.

In addition, analytical modelling using Findley’s power law was also conducted and presented. The reduction factor χ(t) and, ultimately, the time-dependent modulus E(t), were calculated using Findley’s parameters. This work came to the following conclusions:

The differences in short-term properties between the sequences were significant, especially with regards to the ultimate strength of 242.6 MPa for Sequence 1 and 399.05 MPa for Sequence 2, which reflects the fibre arrangement and manufacturing quality despite both using the same manufacturing technique.

Sequence 1 showed a clear increase in the duration of the primary creep stage with higher loads, unlike Sequence 2 which did not exhibit a clear trend. This emphasizes the importance of choosing an appropriate length for creep tests, including to use of an additional second stage.

The creep behaviour was modelled successfully using Findley’s power law, proving the reliability of this approach to simulate the viscoelastic response of pGFRP laminates. As expected, the *n* parameter was almost constant across different stress levels for each sequence, while the *m* parameter increased with the higher loads. Moreover, a general Findley equation was successfully developed for each sequence which provided accurate predictions for strains when compared to the experimental data.

The time-dependent reduction factor χ(t) was calculated at different time intervals for both sequences to predict the reduced flexural modulus. Sequence 1 showed a 23% reduction after 50 years, noticeably different compared to Sequence 2, which was expected to lose about 10%. These results proved that the stacking implemented sequences have an effect on pGFRP material properties and creep performance.

## Figures and Tables

**Table 1:** Material properties of Sequence 1 and Sequence 2.

Properties	Sequence 1	Sequence 2
Density (g/m^3^)	1.80	2.01
Young’s Modulus, $E_{x}/E_{y}$ (MPa)	9530/4800	12,130/5100
Shear Modulus, (MPa)	4000	4280
Compressive Strength, $\sigma_{x}/\sigma_{y}$ (MPa)	150/65	320/76
Tensile Strength, $\sigma_{x}/\sigma_{y}$ (MPa)	321/80	429/100
Fibre volume fraction, $V_{f}$ (%)	61.95	70.45

**Table 2:** Summary of the short-term material properties.

Sequence	Ultimate Strength (MPa)		Ultimate Strain (mm/mm)		Flexural Modulus (MPa)	
	$\sigma_{max}$	Average	$\varepsilon_{ult}$	Average	E	Average
Sequence 1	248.3	242.6	0.02196	0.02289	18,706	17.879
	236.7		0.02051		17,158	
	242.9		0.02209		17,773	
Sequence 2	407.9	399.05	0.02171	0.02197	23,003	21.880
	391.9		0.02173		20,914	
	397.3		0.02248		21,725	

**Table 3:** Summary of the ultimate flexural load and creep load levels.

Sequence	Ultimate Flexural Load (N)	Load Level	Applied Load (N)
Sequence 1	1082	12%	129.8
		24%	259.7
		37%	400.3
Sequence 2	2192	12%	263.0
		24%	526.1
		37%	811.0

**Table 4:** Findley’s power law parameters, as obtained from experimental data fitting.

Sequence	Load Level	$\varepsilon_{o}\;(\%)$	m	n	$E_{t}$ =σ/m (GPa)
Sequence 1	12%	0.10207	0.00627	0.1749	531.0
	24%	0.23399	0.01230	0.1801	541.8
	37%	0.42400	0.01554	0.1623	661.0
Sequence 2	12%	0.15428	0.00702	0.1415	706.1
	24%	0.35350	0.00742	0.1324	1336.0
	37%	0.64820	0.01071	0.1319	1427.3

**Table 5:** Predicted reduction factor and flexural modulus for each sequence.

Time (Years)	Sequence 1		Sequence 2	
	χ(t)	E(t) (MPa)	χ(t)	E(t) (MPa)
1	0.87	15,619.1	0.94	20,624.1
5	0.84	15,000.3	0.93	20,323.7
10	0.82	14,695.2	0.92	20,176.0
50	0.77	13,893.2	0.90	19,785.4

## Data Availability

Not applicable.
